# Supplementary material for: Rational Design of Oxazolidine-Based Red Fluorescent pH Probe for Simultaneous Imaging Two Subcellular Organelles
Source: Biosensors (Basel). 2022 Aug 29;12(9):696. doi: 10.3390/bios12090696 (PMC9496385; doi:10.3390/bios12090696)
Supplement: Supplementary file 1 [file biosensors-12-00696-s001.zip › biosensors-1870142-supplementary.pdf]

biosensors

*Article*

# Rational Design of Oxazolidine-Based Red Fluorescent pH Probe for Simultaneous Imaging Two Subcellular Organelles

**Chunfei Wang [1], Hengyi Fu [1], Jingyun Tan [1], Xuanjun Zhang [1,2,*]**

[1] Faculty of Health Sciences, University of Macau, Macau SAR, 999078, China
[2] MOE Frontiers Science Centre for Precision Oncology, University of Macau, Macau SAR 999078, China
* Correspondence: xuanjunzhang@um.edu.mo (X.Z.).

## Table of contents

**Table S1.** Calculated excitation energy (eV), oscillator strengths and major contribution for **BP** and **BP**$^+$.

**Figure S1**. $^1$H NMR spectrum of **1** in DMSO.

**Figure S2.** $^1$H NMR spectrum of **2** in Acetone.

**Figure S3.** $^{13}$C NMR spectrum of **2** in Acetone.

**Figure S4.** $^1$H NMR spectrum of **BP** in $CDCl_3$.

**Figure S5.** $^{13}$C NMR spectrum of **BP** in $CDCl_3$.

**Figure S6.** HRMS spectrum of **BP**.

**Figure S7.** Plot curve of fluorescence intensity of 10 μM probe **BP** versus pH. The information of the curve: Model, Boltzmann. Equation, $y=A2+(A1-A2)/(1+\exp((x-x_0)/dx))$. $pKa=x_0$.

**Figure S8.** Frontier molecular orbital energy diagram of **BP** and its opening structure **BP**$^+$ in vacuum. Transition energies were calculated using the TD-B3LYP method with 6-31G basis sets.

**Figure S9.** Viabilities of HeLa and U87 cells after incubation with different concentrations of **BP** for 24 h.

**Table S1.** Calculated excitation energy (eV), oscillator strengths and major contribution for **BP** and **BP⁺**.

| Compounds | Energy Gap (eV) | f | Composition | HOMO (eV) | LUMO (eV) |
|---|---|---|---|---|---|
| BP | 2.3207 | 0.3385 | HUMO→LUMO 0.6733 | -0.1206 | 0.1584 |
| BP+ | 1.9704 | 0.1072 | HUMO→LUMO 0.7063 | 0.1526 | 0.6844 |

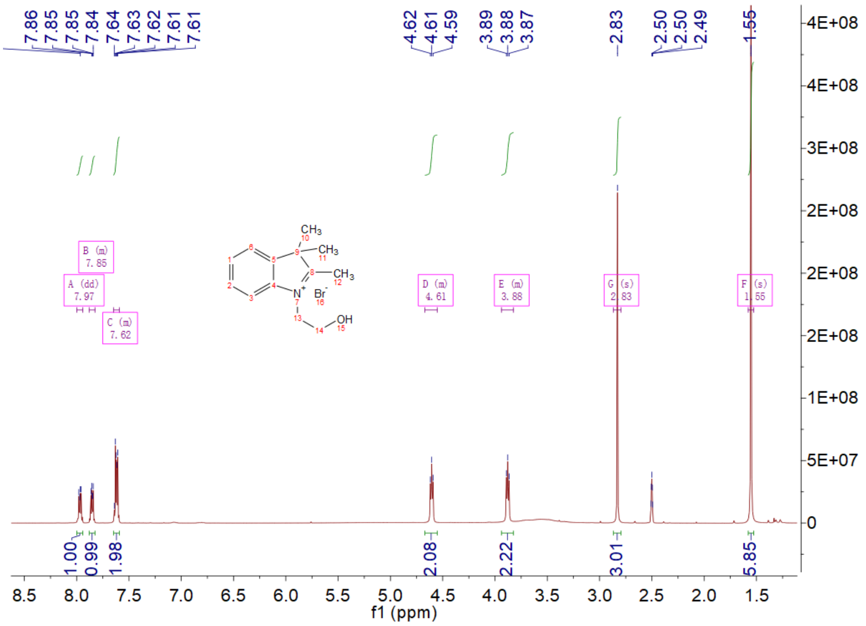


**Figure S1.** ¹H NMR spectrum of **1** in DMSO.

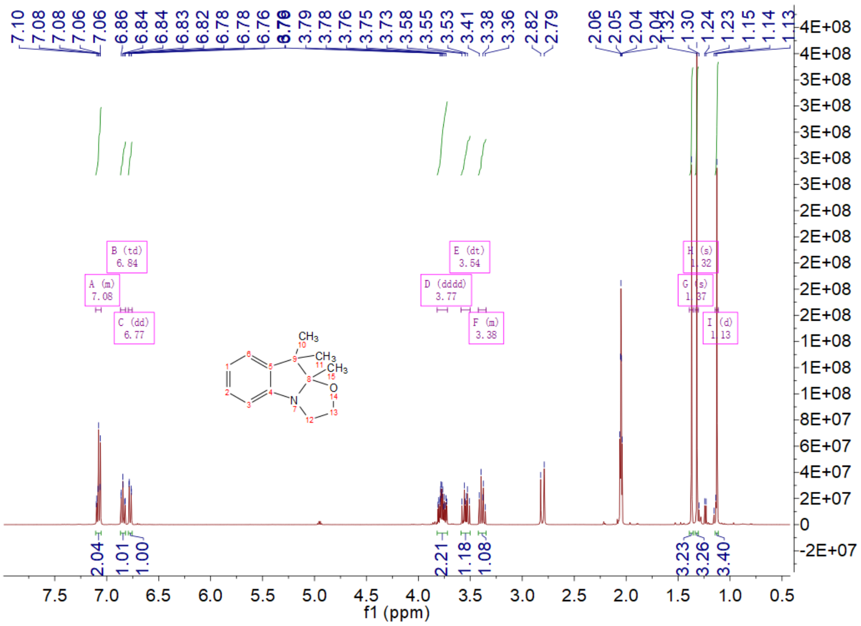


**Figure S2.** ¹H NMR spectrum of **2** in Acetone.

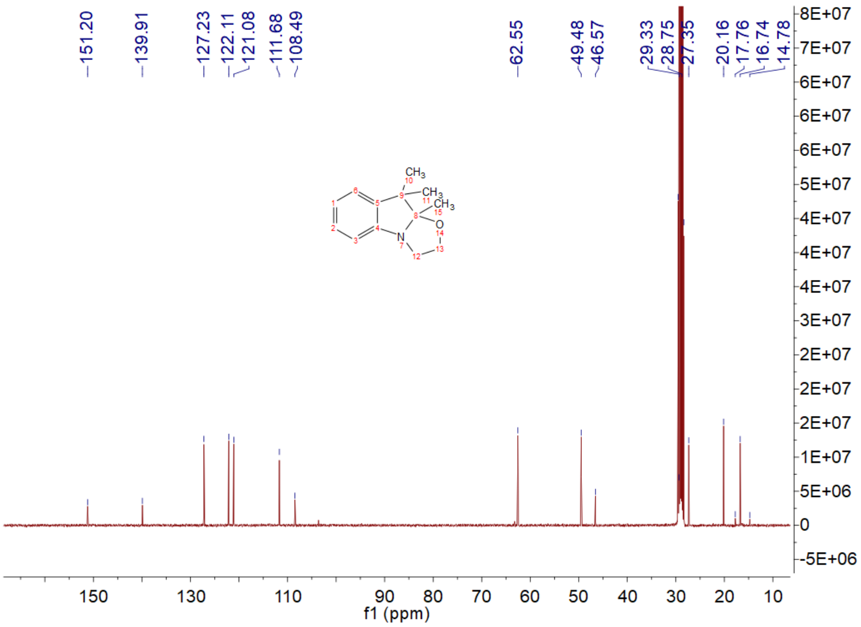

**Figure S3.** $^{13}$C NMR spectrum of **2** in Acetone.

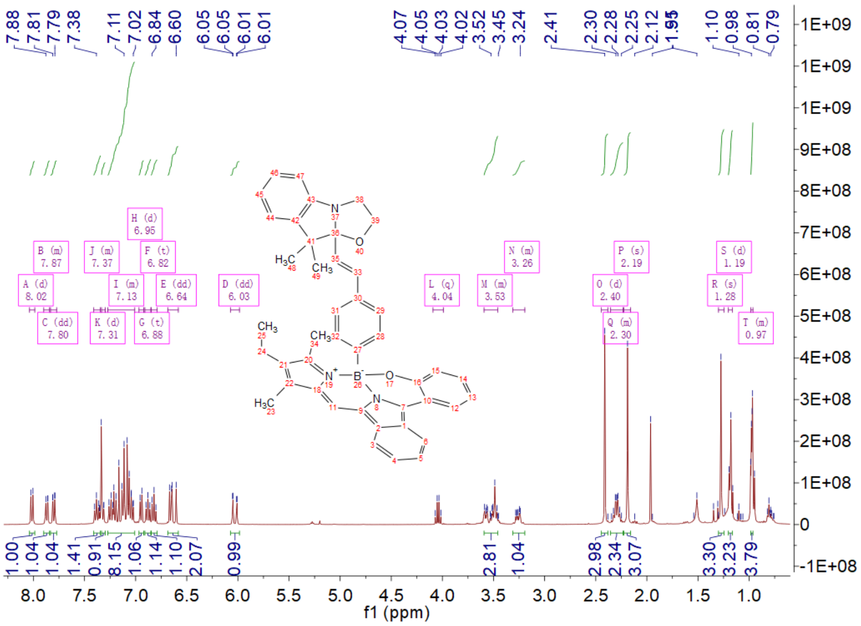

**Figure S4.** $^{1}$H NMR spectrum of **BP** in $CDCl_3$.

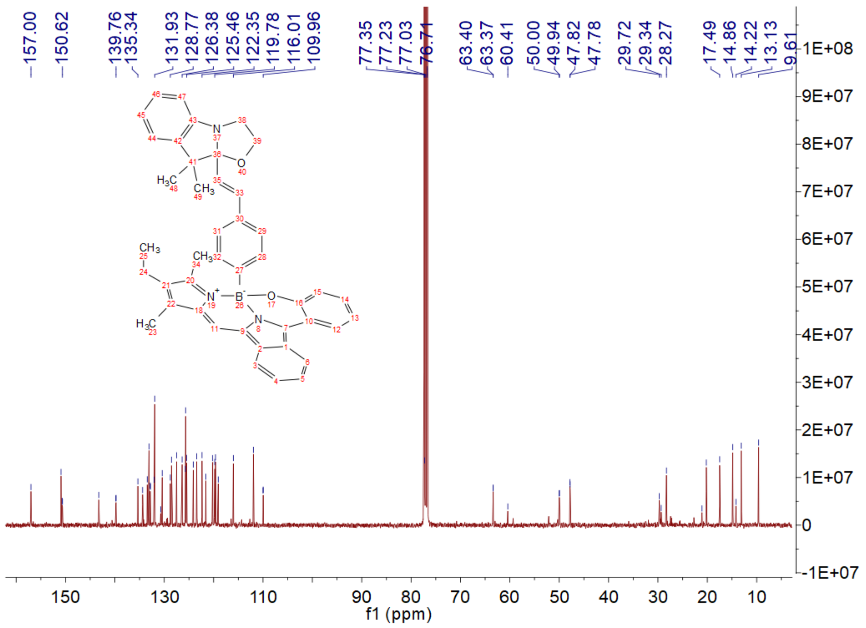

**Figure S5.** $^{13}C$ NMR spectrum of **BP** in $CDCl_3$.

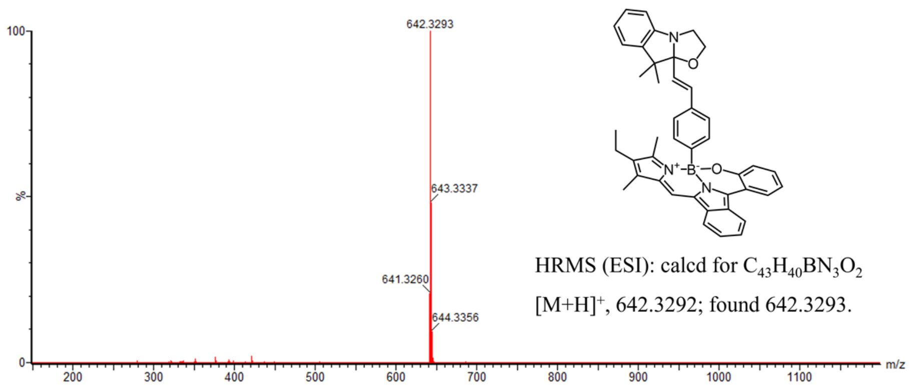


**Figure S6.** HRMS spectrum of **BP**.

*1. Calculation of pKa Value.*

The fluorometric titration as a function of pH was obtained fluorescence spectra. The equation below was used to calculate the pKa value of probe BP [1].

$$F=(Fmin[H+]n + FmaxKa)/(Ka+[H+]n)$$

The expression of the steady-state fluorescence intensity F as a function of the proton concentration has been extended for the case of n: complex between H+ and a fluorescent dye. Where Fmin and Fmax are the fluorescence intensities at maximal and minimal H+ concentrations, respectively. n is apparent stoichiometry of H+ binding to the probe BP. Nonlinear fitting of equation expressed above to the fluorescence titration data was plotted as a function of H+ concentration.

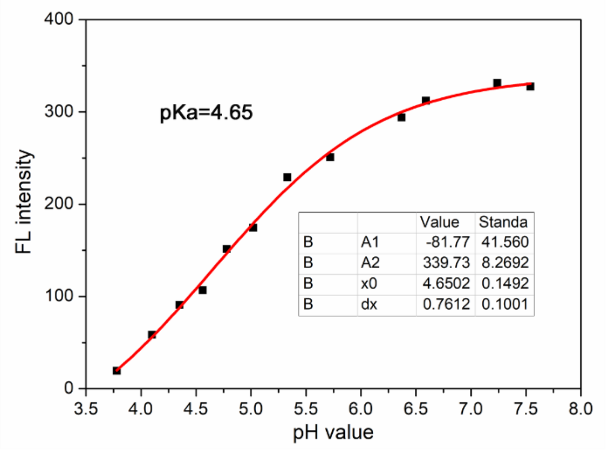


**Figure S7.** Plot curve of fluorescence intensity of 10 μM probe **BP** versus pH. The information of the curve: Model, Boltzmann. Equation, y=A2+(A1-A2)/(1+exp((x-x0)/dx)). pKa=x0.

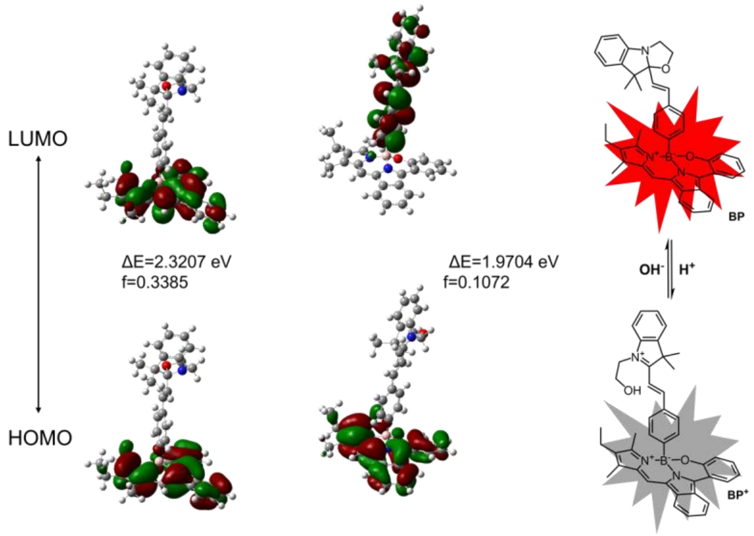


**Figure S8.** Frontier molecular orbital energy diagram of **BP** and its opening structure **BP⁺** in vacuum. Transition energies were calculated using the TD-B3LYP method with 6-31G basis sets.

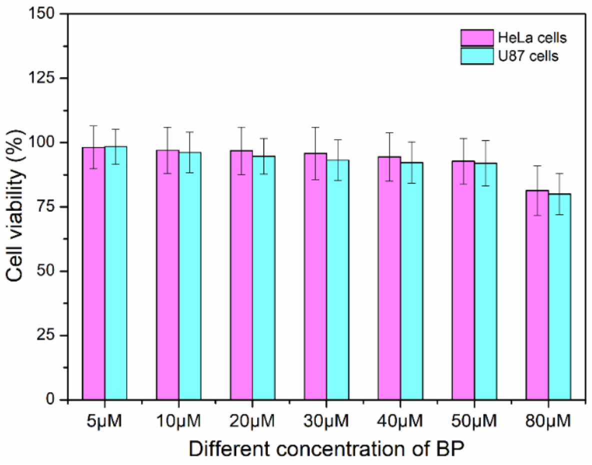


**Figure S9.** Viabilities of HeLa cells and U87 cells after incubation with different concentrations of **BP** for 24 h.

**Reference**

[1] Mazi, W.; Yan, Y.; Zhang, Y.; Xia, S.; Wan, S.; Tajiri, M.; Luck, R. L.; Liu, H., A near-infrared fluorescent probe based on a hemicyanine dye with an oxazolidine switch for mitochondrial pH detection. *J. Mater. Chem. B* **2021,** 9, (3), 857-863.
